# Reply to de Jong, M.A.; Thomeer, H.G.X.M. The Use of Multifrequency Tympanometry in Ménière’s Disease. Comment on “Tsilivigkos et al. Can Multifrequency Tympanometry Be Used in the Diagnosis of Meniere’s Disease? A Systematic Review and Meta-Analysis. *J. Clin. Med.* 2024, *13*, 1476”

**DOI:** 10.3390/jcm14072286

**Published:** 2025-03-27

**Authors:** Christos Tsilivigkos, Evangelos Ν. Vitkos, Eleftherios Ferekidis, Athanasia Warnecke

**Affiliations:** 1 First Department of Otolaryngology, Hippokration General Hospital, National and Kapodistrian University of Athens, 15772 Athens, Greece; drferekidis@gmail.com; 2 Department of Oral and Maxillofacial Surgery, George Papanikolaou General Hospital, 56429 Thessaloniki, Greece; 3 Department of Otorhinolaryngology—Head and Neck Surgery, Hannover Medical School, 30625 Hanover, Germany; 4 Cluster of Excellence Hearing4all, German Research Foundation, 30625 Hannover, Germany

We would like to extend our gratitude to Dr. de Jong and Dr. Thomeer for their thoughtful comments on our previous article discussing the potential clinical application of multifrequency tympanometry in diagnosing Ménière’s disease (MD) [1]. MD remains a clinical entity with significant diagnostic challenges and is often a diagnosis of exclusion. Therefore, a simple and rapid clinical test, such as multifrequency tympanometry, could serve as a valuable adjunct in evaluating patients with MD.

We agree with our colleagues that at the resonant frequency of a mechanical system, admittance (Y) and conductance (G) are equal. Furthermore, tympanometric measurements at 2 kHz are more sensitive in detecting alterations in cochlear pressure and the annular ligament [2,3]. It is also important to note that admittance and conductance are not equivalent at 2 kHz and thus should not be directly compared at this frequency.

The suggestion that G-width at 2 kHz may better highlight the presence of endolymphatic hydrops (EHs) rather than MD is noteworthy, as G-width reflects shifts in cochlear pressure. This observation may explain why increased G-width at 2 kHz has also been observed in healthy individuals, who may have asymptomatic EH. Although the exact prevalence of asymptomatic EH is unclear, a study by Kimura et al. reported that approximately 7% of healthy individuals may exhibit EH without any symptoms of MD [4]. Therefore, false positives in healthy individuals could be attributed to this phenomenon.

Given the overlap of positive results in MD patients and healthy individuals, we recommend that clinicians interpret multifrequency tympanometry results with caution. However, when used in combination with medical history, clinical examination, and pure-tone audiometry, multifrequency tympanometry may be a useful tool in the neurotological department. As we have stated in our original article, multifrequency tympanometry could be valuable in diagnosing ears at an increased risk of disease in patients with unilateral MD.

Lastly, the aetiology of MD is still not fully understood. Further research should aim to elucidate the pathophysiology of this complex condition. We welcome further discussion on this topic, since the currently available neurotological tests cannot directly evaluate the underlying pathophysiological mechanisms of MD.
